# ME/CFS and Long COVID share similar symptoms and biological abnormalities: road map to the literature

**DOI:** 10.3389/fmed.2023.1187163

**Published:** 2023-06-02

**Authors:** Anthony L. Komaroff, W. Ian Lipkin

**Affiliations:** ^1^Department of Medicine, Brigham and Women’s Hospital, Harvard Medical School, Boston, MA, United States; ^2^Center for Infection and Immunity, Mailman School of Public Health, Vagelos College of Physicians and Surgeons of Columbia University, New York, NY, United States

**Keywords:** chronic fatigue syndrome (CFS), myalgic encephalomyelitis (ME), myalgic encephalomyelitis/chronic fatigue syndrome (ME/CFS), Long COVID, post-acute sequelae of SARS-CoV-2 infection, post-infectious fatigue syndrome, post-intensive care unit syndrome

## Abstract

Some patients remain unwell for months after “recovering” from acute COVID-19. They develop persistent fatigue, cognitive problems, headaches, disrupted sleep, myalgias and arthralgias, post-exertional malaise, orthostatic intolerance and other symptoms that greatly interfere with their ability to function and that can leave some people housebound and disabled. The illness (Long COVID) is similar to myalgic encephalomyelitis/chronic fatigue syndrome (ME/CFS) as well as to persisting illnesses that can follow a wide variety of other infectious agents and following major traumatic injury. Together, these illnesses are projected to cost the U.S. trillions of dollars. In this review, we first compare the *symptoms* of ME/CFS and Long COVID, noting the considerable similarities and the few differences. We then compare in extensive detail the underlying *pathophysiology* of these two conditions, focusing on abnormalities of the central and autonomic nervous system, lungs, heart, vasculature, immune system, gut microbiome, energy metabolism and redox balance. This comparison highlights how strong the evidence is for each abnormality, in each illness, and helps to set priorities for future investigation. The review provides a current road map to the extensive literature on the underlying biology of both illnesses.

## Introduction

Shortly after the onset of the COVID-19 pandemic, it became clear that some patients remained unwell for months to years after “recovering” from the acute infection. They suffered from fatigue, cognitive problems, headaches, disrupted sleep, myalgias and arthralgias, post-exertional malaise, orthostatic intolerance, tachyarrhythmias and gastrointestinal complaints, all of which greatly interfered with their ability to function at home and at work. In this post-COVID period, the most severely affected people were housebound and disabled. This was true even of people who had not been severely ill with acute COVID-19. The illness has been given several names, including post-acute SARS-CoV-2 sequelae (PASC), post-COVID-19 condition (PCC), post-acute COVID-19 syndrome (PACS), and “Long COVID”—the name we use in this review. We distinguish it from other post-COVID conditions in Table 1, below.

**TABLE 1:** Post-acute sequelae of SARS-COV-2 infection (PASC).

1. *Tissue injury of multiple organs*, including lungs (9), brain (10, 11), heart (12), kidneys (13), and gut (14) that may be visible with imaging technologies, and that has the potential to lead to long-term organ dysfunction.
2. *New onset of major diseases* including diabetes mellitus, cardiovascular diseases (including myocardial infarction), stroke and pulmonary failure (incidence increased by 150–400%), and a greatly increased risk of death (160%) in the first year following acute COVID-19, compared to matched populations that did not develop COVID-19 (12, 15, 16).
3. *Long COVID:* a group of *persisting symptoms* that interfere with the ability to function at home or at work, are not explained by major organ injury (e.g., pulmonary fibrosis, cardiomyopathy, cerebral infarction, kidney failure). These symptoms include fatigue, cognitive problems, headaches, disrupted sleep, myalgias and arthralgias, post-exertional malaise, orthostatic intolerance, tachyarrhythmias and gastrointestinal complaints such as diarrhea. Although there is no major organ injury, there are some underlying biological abnormalities which are summarized in this review. People with Long COVID often are referred to as “long haulers.”

In this review, we compare the symptoms of Long COVID and ME/CFS, noting considerable similarities and some differences. Early in the study of both illnesses, the lack of objective biomarkers led some to question whether the illnesses were “real”—whether people might be imagining or even fabricating their symptoms.

In this review we summarize the emerging evidence that, in fact, there are many underlying biological abnormalities reported in both illnesses, documented by multiple laboratories. Moreover, we show that the two illnesses *share* many of these underlying abnormalities, just as they share many symptoms. That is, both illnesses are “real,” and both share similar biological abnormalities.

Understanding the underlying biology of these illnesses is critically important, given the burden they are placing on all societies. The National Academy of Medicine and Centers for Disease Control and Prevention (CDC) estimate that, in the U.S., ME/CFS affects up to 2.5 million people and generates direct and indirect expenses of approximately $17–24 billion annually (1). It also has been reported in many countries around the world.

Post-COVID-19 conditions may affect 65 million people, globally (2). In the U.S., nearly 2% of the total civilian labor force is unable to work because of these illnesses, with foregone wages of $170–230 billion annually (3). Senior economists have estimated that the aggregate cost of medical care, lost productivity and disability may be $3.7 trillion over the next 5 years (4, 5). The most important, and unanswered, question regarding the economic impact of Long COVID is how long it will last. If, as with most people with ME/CFS, it lasts decades, the burden will be even greater than this projection. Because of the exceptional anticipated burden, the National Institutes of Health has dedicated over $1 billion in support of studies of the underlying biology and natural history of various post-COVID conditions.

In Tables 4–7 we try to efficiently summarize the findings from the large body of ME/CFS and post-COVID-19 literature. Our intention is to provide both an overview to general readers and a road map to a very large literature for investigators interested in pursuing the role of specific abnormalities in either or both illnesses. In summarizing what is known about biological abnormalities in both illnesses, we have cited both the “positive” studies that find an abnormality and the “negative” ones that do not. This allows readers to assess the strength (or weakness) of a reported finding.

As in any review, the studies we include are of varying size and quality. It is our hope that the road map we provide will help all interested readers judge for themselves how solid the evidence is for each of the underlying biological abnormalities, in each of the two illnesses, and where holes exist in our knowledge that need to be filled.

## Brief summary of the illnesses

### Overview of different conditions following acute COVID-19

Most people recover completely from *acute* COVID-19. However, others develop a variety of different post-acute sequelae of SARS-CoV-2 infection (PASC). The terminology for these different endotypes has not been standardized, in part because the detailed longitudinal studies necessary to generate the empirical data by which to define and distinguish different PASC endotypes have not yet been completed.

In Table 1, we propose three broad categories of PASC. Although we mention the first two categories for completeness, this review discusses *only* the third category—the condition called Long COVID. Some authorities refer to Long COVID as “PASC.” We think that is imprecise, since the tissue injury to multiple organs and the new onset of major diseases listed in Table 1 surely also are “sequelae” of COVID-19. In any event, the data summarized in Tables 3–7 of this review refer only to Long COVID.

The symptoms of Long COVID limit an individual’s ability to function at home or at work. The CDC states that in the “post-COVID condition” (which we take as equivalent to “Long COVID”) symptoms can be present four or more weeks after infection with SARS-CoV-2. The World Health Organization defines the condition as the continuation or development of new symptoms 3 months after the initial SARS-CoV-2 infection and lasting for at least 2 months with no other explanation.

Some evidence indicates that Long COVID is more likely to develop in: people who are sickest and have the greatest evidence of inflammation with acute COVID-19; were PCR positive; are female; and have a premorbid history of asthma, chronic obstructive pulmonary disease and depression. However, many people with Long COVID have none of these risk factors (6–8).

### Long COVID

How frequently does Long COVID follow acute COVID-19? A wide range of values for the incidence of Long COVID following infection with SARS-CoV-2 has been reported, depending on how the investigators have defined Long COVID and how rigorously they have pursued alternative explanations for the persisting symptoms.

A meta-analysis of 57 studies involving over 250,000 people found that ongoing symptoms impairing functional mobility persisted in 43% of people with acute COVID-19 for at least *6 months* after acute infection (511). A study of nearly 100,000 cases and matched never-infected controls found that over 40% of cases remained with persistent symptoms and impairment across all daily activities at *18 months* (8). A study of six thousand hospitalized patients compared to uninfected matched controls found persistent symptoms in approximately 30% of cases—much more often than in uninfected controls—at least *2 years* after acute infection (512). Among people with persistent, debilitating symptoms following acute COVID-19, an estimated 13–45% meet the National Academy of Medicine case definition for ME/CFS (447, 513–515).

### ME/CFS

Interest in ME/CFS (originally called “chronic fatigue syndrome”) surged in the mid-1980s, although a very similar illness has been described in the medical literature for several centuries. In the 19th and early 20th centuries the name “neurasthenia” was used to refer to a similar illness (516, 517). Since the 1980s, the names “chronic fatigue syndrome” and “systemic exertional intolerance disease” have been used. The illness also has several case definitions. We prefer the one proposed by the U.S. National Academy of Medicine (1), that is summarized in Table 2.

**TABLE 2:** U.S. National Academy of Medicine case definition for ME/CFS.

Substantial impairment in the ability to function at home or at work, lasting for more than 6 months, accompanied by profound fatigue, of new or definite onset (not lifelong), not substantially alleviated by rest AND
Post-exertional malaise AND
Unrefreshing sleep PLUS at least one of: Cognitive impairment OR Orthostatic intolerance _______
**Definitions:**
*Post-exertional malaise (PEM):* A prolonged exacerbation of a patient’s baseline symptoms after physical/cognitive/orthostatic exertion or stress. It may be delayed relative to the trigger. *Unrefreshing sleep:* Feeling unrefreshed after sleeping many hours. *Cognitive impairments:* Problems with thinking exacerbated by exertion, effort, or stress or time pressure. *Orthostatic intolerance:* Symptoms worsen upon assuming and maintaining upright posture and are improved, though not necessarily abolished, by lying back down or elevating feet.
*U.S. National Academy of Medicine* (1).

Myalgic encephalomyelitis/CFS often, but not always, follows in the wake of an apparent “infectious-like” illness characterized by respiratory and gastrointestinal symptoms, fatigue, myalgias and other symptoms as well as fever and lymphadenopathy (518). This “infectious-like” illness often is little different, initially, from the common, transient infectious illnesses that most people experience throughout life. It is not standard medical practice to test for the responsible infectious agent in people with common and transient infectious illnesses. Thus, typically no testing has been done to determine the cause of the initial “infectious-like” illness that then becomes a chronic illness in subsequent months and years.

Although in this review we are describing cases of ME/CFS that occur sporadically, there also have been apparent epidemics of a similar illness described in communities (141, 519–523) and in large hospitals over the past century (524). In one report, 6% of a community of 2,500 people—most young or middle-aged adults—became suddenly ill with an infectious-like illness followed by at least 5 months of a cyclic, debilitating illness characterized by fatigue, cognitive impairment (confirmed on objective testing) and pain (520). Details on symptoms, physical examination findings and laboratory test results in these studies are not sufficient to determine how similar these illnesses were to ME/CFS.

The severity of the symptoms, and the functional impairment, can range widely from one person to another. The functional impairment in people with ME/CFS may be even greater than in those with congestive heart failure and major depression (525, 526), and greater than in those with Long COVID (527). Some people remain able to fulfill their main responsibilities at work and at home, although hobbled. Others are bed-ridden or housebound, and unable to work. For most individuals with ME/CFS the symptoms are cyclic, with some relatively “good” days and frequent “bad” days. Several stressors—exercise, prolonged upright position, cognitive and emotional upset—typically produce a worsening of all of the symptoms of the illness. This condition, called post-exertional malaise, is a cardinal feature of the illness (1).

Attempts to identify a single and possibly novel infectious agent as the cause of most cases of ME/CFS have been unsuccessful. For example, claims that murine leukemia viruses cause ME/CFS have been refuted (528, 529), as have similar claims for Borna disease virus (530).

Several other conditions often are present in some people with ME/CFS: autonomic dysfunction (particularly postural orthostatic tachycardia syndrome), various rheumatologic conditions (particularly fibromyalgia), neurologic deficits (such as sensory hypersensitivity and small fiber neuropathy), secondary depression and anxiety, new or worsened allergic disorders, and endometriosis (531, 532).

## Comparison of the symptoms

The symptoms reported by people with both ME/CFS and Long COVID have been integrated in a recent meta-analysis of 21 studies (17). In Table 3, we summarize the long list of symptoms shared by these two illnesses (and shared with other illnesses). The cardinal symptom of ME/CFS, post-exertional malaise, also is reported by the vast majority of people with Long COVID (533, 534). As seen in Table 3, most symptoms in the two illnesses are similar, although decreased smell and taste, rash and hair loss are frequent in Long COVID but only rarely reported by people with ME/CFS, and painful lymph nodes, chemical sensitivities and tinnitus are frequent in ME/CFS but rarely reported in Long COVID.

**TABLE 3:** Comparison of symptoms, ME/CFS, and Long COVID.

Symptom	ME/CFS	Long COVID	Symptom	ME/CFS	Long COVID
Fatigue	✓	✓	Poor appetite	✓	✓
Post-exertional malaise	✓	✓	Orthostatic intolerance	✓	✓
Headaches	✓	✓	Palpitations	✓	✓
Sleep disorder	✓	✓	Breathlessness	✓	✓
Impaired reasoning	✓	✓	Nausea and diarrhea	✓	✓
Impaired memory	✓	✓	Chills	✓	✓
Impaired attention	✓	✓	Cough	✓	✓
Secondary depression	✓	✓	Decreased smell and taste		✓
Secondary anxiety	✓	✓	Rash and hair loss		✓
Reduced activity	✓	✓	Painful lymph nodes	✓	
Myalgia/arthralgia	✓	✓	Chemical sensitivities	✓	
Muscle weakness	✓	✓	Tinnitus	✓	
Hot and cold spells	✓	✓			

ME/CFS, myalgic encephalomyelitis/chronic fatigue syndrome. Adapted from: Wong DJ (17).

## Comparison of the underlying objective biological abnormalities

A wide range of objective abnormalities have been reported in both Long COVID and ME/CFS. Multi-organ magnetic resonance imaging (MRI) imaging of many Long COVID patients and non-symptomatic post-COVID control subjects has revealed abnormalities involving many organs up to 1 year following acute COVID-19, although it is unclear if the *anatomic* MRI abnormalities reflect defective organ *function*: correlation of MRI abnormalities with persistent symptoms was poor (535).

Probably with both illnesses, and certainly with ME/CFS, underlying biological abnormalities appear to change over time. Initially, there is activation of neuroendocrine axes and cytokine production, followed after several years by an apparent “exhaustion” of neuroendocrine and immunologic activity (260, 536).

The most compelling findings thus far have been described in the central and autonomic nervous system, immune system, infectious agents, metabolism, and the cardiopulmonary system. We will focus on those abnormalities in this review.

### Central and autonomic nervous system

Table 4 summarizes the different abnormalities that have been identified in the central nervous system (CNS) and autonomic nervous system (ANS) in ME/CFS and in Long COVID. Most of them have been reported by multiple laboratories and are common to both illnesses. Often, the measured abnormality has been shown to be correlated with the presence and severity of symptoms. We comment in the text only on those abnormalities where the evidence is most extensive.

**TABLE 4:** Neurologic/neuromuscular abnormalities.

Finding	ME/CFS	Long COVID
**Cognition**		
Cognitive deficits	*Positive studies:* deficits primarily in attention and reaction time (18–32) Deficits worsen after physical and cognitive exertion (33), and are not explained by concomitant mood disorders (19, 20, 34, 35). Functional MRI (fMRI) studies find that people with cognitive deficits have greater activation of brain activity (36, 37), particularly in response to a cognitive challenge (38) fMRI abnormalities are increased following physical or cognitive exertion (39), appear not to worsen with time (40), and are not due to an intent to perform poorly (35, 41). *Negative studies:* some studies have not found cognitive deficits (42, 43).	*Positive studies:* deficits of several types, particularly of sustained attention and processing speed (44–49). Two months after acute infection, more than half of patients with Long COVID have impairment in executive function, memory that correlates with EEG and MRI abnormalities (50, 51). Simultaneous study of ME/CFS and Long COVID patients shows similar deficits in attention and processing speed, somewhat worse in ME/CFS (49). Scoping review of 25 studies of cognition in Long COVID found abnormalities in memory (delayed recall and learning), attention, and executive functions (e.g., abstraction, set shifting) appeared to be the most affected domains (48). Deficits are worst in those who were most severely ill with acute COVID-19 (46). SARS-CoV-2 infection of brain organoids leads to destruction of synapses (52).
**Neurovascular abnormalities**		
Reduced cerebral blood flow (CBF)	*Positive studies:* demonstrated by single-photon emission computed tomography (SPECT) (53–59), by MRI with arterial spin labeling (59–64) and by transcranial Doppler flow imaging (65–67). Reduced CBF seen both at rest and following exertional, cognitive or orthostatic challenge, and was not explained by concomitant mood disorders (56–59). Reduced CBF persisted following orthostatic challenge (68) and correlated with symptom severity (59, 61, 62). *Negative studies:* (69–71)	*Positive studies:* reduced CBF from the time of acute infection, and persisting for at least 10 months (72–74).
**Neuroendocrine abnormalities**		
Down-regulation of the hypothalamic-pituitary-adrenal (HPA) axis	*Positive studies:* reduced levels of corticotropin-releasing hormone (CRH), adrenocorticotropic hormone (ACTH) and circulating, urinary or salivary cortisol (75–81). HPA axis abnormalities may be secondary to redox imbalance (82). HPA abnormalities confirmed in two meta-analyses (83, 84). *Negative studies:* (85, 86).	*Positive studies:* similar abnormalities (7, 87–91). *Negative studies:* (92).
Hypothalamic-pituitary-prolactin axis abnormalities	*Positive studies:* increased prolactin response to serotonin-releasing agents (buspirone and D-fenfluramine), due to elevated activity of pre-synaptic serotonergic neurons (93–97).	
Hypothalamic-pituitary-growth hormone axis abnormalities	*Positive studies:* reduced basal levels of IGF-1 and IGF-2 and growth hormone, and blunted growth hormone responses to hypoglycemic challenge (98–101).	*Positive studies:* similar findings (87–89).
Hypothalamic-pituitary-thyroid hormone axis abnormalities	*Positive studies:* (102)	
**Hypometabolic state**		
Hypometabolism in brain	*Positive studies:* hypometabolism in multiple brain areas demonstrated by PET and magnetic resonance spectroscopy (103, 104).	*Positive studies:* similar abnormalities (44, 105–108). May sometimes be accompanied by amyloid-beta deposition (108). Can last 10–12 months after acute COVID (106, 108). Cognition may improve over the first year (109). Correlates with the duration of neurocognitive symptoms post-COVID (107, 109). Found both children and adults (110). *Negative studies:* (111)
**Autonomic dysfunction**		
Autonomic dysfunction	*Positive studies:* a variety of autonomic testing has revealed abnormalities (112–127). The presence of these abnormalities has been confirmed in a meta-analysis (122) and correlate with symptoms (140). *Negative studies:* (56, 128)	*Positive studies:* similar testing technologies have revealed similar abnormalities (72, 73, 126, 129–140).
**Gray and white matter abnormalities on imaging**		
Increased signal in white matter on magnetic resonance imaging (MRI)	*Positive studies:* (53, 141–146) MRI abnormalities are negatively associated with physical functioning (147). *Negative studies:* (42, 148)	*Positive studies:* (51, 149, 150) MRI abnormalities correlate with measured cognitive impairment (50, 51, 149–151).
Reduced white matter volume on MRI	*Positive studies:* (152–154) which is progressive (155)	*Negative studies:* (50)
Altered gray matter volume on MRI	*Positive studies (reduced gray matter volume):* (153, 154, 156, 157) Reduced gray matter volume correlates with symptoms (155, 158).	*Positive studies (increased volume early, decreased volume later and/or when more severe):* Gray matter abnormalities (74, 151, 159–161) are dynamic on longitudinal studies: initial increase in gray matter in limbic areas early in disease is followed by atrophy, particularly in those with more severe acute disease (74).
**Pain/sensory abnormalities**		
Lowered threshold for pain in response to various stimuli	*Positive studies:* (162–167) Lowered threshold exacerbated by orthostatic stress (168), enhanced by concomitant redox imbalance (169, 170) and possibly influenced by differential expression of specific microRNAs (171). Whether this “central sensitization” is a core element of the illness or an epiphenomenon remains uncertain (172).	*Positive studies:* symptom inventory suggests central sensitization, but pain threshold has not been directly measured (173).
Impaired response to cognitive, motor, visual and auditory challenge on functional MRI	*Positive studies:* (36–38, 174–179)	
Small fiber neuropathy	*Positive studies:* (180)	*Positive studies:* (72, 181–184)
**Electroencephalographic abnormalities**		
Abnormal electroencephalogram	*Positive studies:* spectral analysis pattern that distinguishes people with ME/CFS from people with major depression and from matched healthy control subjects (185).	*Positive studies:* higher regional current density and connectivity at delta band, and abnormal spindles, up to 12 months later (50, 186).
**Sleep abnormalities**		
Sleep disorders	*Positive studies:* non-restorative sleep, restlessness, insomnia, sleep apnea more common in people with ME/CFS (187), although formal sleep laboratory studies have produced inconsistent results (188, 189). Meta-analysis of studies of ˜1300 adults and adolescents finds longer sleep latency, reduced sleep efficiency, increased longer REM latency, longer bedtime, and altered sleep microstructure (190)	*Positive studies:* chronic insomnia, hypersomnia, irregular sleep patterns, associated with mood disorders (191–196).
**Muscle abnormalities**		
Muscle abnormalities	*Positive studies:* oxidative and nitrosative stress, mitochondrial and bioenergetic dysfunction, as reviewed in detail elsewhere (197). Decreased muscle mass action potential (M-wave amplitude and duration) (198) and impaired energy metabolism (201). Disturbed muscle membrane function in motor units, demonstrated by nerve conduction studies (199), and possibly secondary to autonomic dysfunction (200).	*Positive studies:* Decreased muscle mass action potential (M-wave amplitude and duration) (198).
**Neurometabolic/neuroinflammatory/neuroimmunologic abnormalities**		
Metabolic dysfunction of glial cells and neuroinflammation seen on positron emission tomography (PET), magnetic resonance spectroscopy (MRS) or pathology	*Positive studies:* demonstrating widespread activation of both astrocytes and microglia (103, 202–204). *Negative studies:* (205)	*Positive studies:* (150, 206, 207)
Autoantibodies against neural central and autonomic targets	*Positive studies:* autoantibodies against neural targets that correlate with the presence and severity of symptoms in some studies (208–218).	*Positive studies:* (219)
**Miscellaneous neurological abnormalities**		
Impaired connectivity	*Positive studies:* (220–228) Impaired connectivity correlates with severity of symptoms (221, 222, 224). Impaired connectivity may be particularly prevalent in the brainstem (120, 229). *Negative studies:* (230)	*Positive studies:* (50, 149, 206)
Cerebrospinal fluid (CSF) abnormalities	*Positive studies:* (231, 232), particularly increased levels of proteins (e.g., α2-macroglobulin, keratin 16, orosomucoid) indicating tissue injury and repair (231).	*Positive studies:* during acute infection, frequent blood-brain barrier dysfunction; elevated protein, lactate and cytokine levels; elevated lymphocyte and monocyte count (233). Although autopsy study finds viral RNA throughout the brain many months later (11), no viral RNA found in CSF (233, 234). Elevated CSF cytokine levels remain elevated for months (233).
Abnormal transient receptor potential ion channel	*Positive studies:* (235, 236)	*Positive studies:* (237)
Brainstem dysfunction	*Positive studies:* (229, 238) Brainstem volume changes on MRI (239)	*Positive studies:* synthesis of evidence incriminates brainstem dysfunction (240) Brainstem volume changes on MRI (239).
Plasma markers of neural injury		*Positive studies:* (241)

#### Cognition

Psychometric testing has revealed cognitive deficits in both illnesses. In people with ME/CFS, testing typically has been performed years after the illness began: there have been few longitudinal studies of the trajectory of cognitive deficits. In contrast, because the inciting infectious agent is clear in Long COVID, and because longitudinal studies were launched shortly after the onset of the pandemic, it is clear that the initial cognitive deficits can persist for many months. One study found there may be some improvement by the end of the first year (537).

With both illnesses, the most consistently observed abnormalities have been impaired attention and information processing speed. Long-lasting objective cognitive impairment has been demonstrated even in people with mild acute COVID-19 (538, 539). One study estimated that the measured cognitive deficit was equivalent to 10 years of aging (540). Measured cognitive impairment correlated significantly with abnormalities seen with EEG and MRI studies (50, 51).

The most persuasive evidence that the persistent cognitive deficits in Long COVID reflect damage to the CNS comes from a large population-based longitudinal study in which MRI scans and cognitive testing were performed both before the pandemic and after. Compared to a matched group that did not develop COVID-19, those who developed COVID-19 (even those not requiring hospitalization) had greater reduction in gray matter thickness and in global brain size, and greater cognitive decline (541).

#### Neurovascular abnormalities

In both illnesses, there is a reduction in cerebral blood flow. This likely results from autonomic dysfunction (Table 4) and possibly from reduced blood volume (Table 7). It may contribute to some of the symptoms of both illnesses, including fatigue and impaired cognition; it may also reflect reduced physical activity and reduced oxygen consumption (hypometabolism).

#### Neuroendocrine abnormalities

The first neurological abnormalities to be documented in ME/CFS were a group of different neuroendocrine abnormalities. In particular, there appears to be down-regulation of activity in several hypothalamic-pituitary axes (Table 4) in ME/CFS, with some similar reports in Long COVID (542). These neuroendocrine changes could have bidirectional interactions with various immunologic and vascular abnormalities that also are seen in both illnesses (542).

#### Autonomic nervous system

Many of the common symptoms seen in people with both illnesses could reflect autonomic dysfunction. In addition, autonomic dysfunction could explain other underlying biological disturbances, such as the reported neurovascular abnormalities. The autonomic dysfunction, in turn, could be caused by other biological abnormalities seen in both illnesses, particularly antibodies directed at autonomic receptors (129) and biopsy-documented small-fiber neuropathy (72, 180–182).

As summarized in Table 4, autonomic abnormalities have been well documented in both illnesses. Abnormalities of both the sympathetic and parasympathetic arms of the autonomic nervous system reflect “dyshomeostasis” (130): poor modulation of the balance between the two systems, with the imbalance favoring expression of the sympathetic system (72, 73, 131–134). Objective autonomic dysfunction is common in the first 6–12 months following acute COVID-19, becoming less common by 24 months following infection, in comparison to matched comparison groups without COVID-19 (73).

#### Magnetic resonance imaging (MRI)

Magnetic resonance abnormalities involving both gray matter and white matter have been found in both illnesses and been shown to correlate positively with measured objective cognitive impairment (50, 51, 149, 150). Impaired responses to a variety of challenges have been revealed by functional MRI.

### Immune system and infectious agents

Myalgic encephalomyelitis/CFS often follows in the wake of an “infectious-like” illness. Long COVID (by definition) follows in the wake of acute infection with SARS-CoV-2. As summarized in Table 5, a variety of immunological parameters distinguish people with ME/CFS from healthy control subjects of the same age and gender. The same is true of Long COVID: immune parameters distinguish patients from post-COVID patients who no longer have symptoms, and from non-infected controls subjects. Moreover, many of the *same* immune parameters distinguish patients with ME/CFS and with Long COVID from comparison/control groups.

**TABLE 5:** Immunologic findings and infectious agents.

Finding	ME/CFS	Long COVID
**Immunologic studies**		
Decreased natural killer (NK) cell function, *in vitro*	*Positive studies:* (236, 237, 242–255) *Negative studies:* (256, 257).	*Negative studies:* (258)
Ion channel abnormalities in NK cells	*Positive studies:* (236, 237)	
Increased numbers of NK cells	*Positive studies:* (243, 248, 252) *Negative studies:* (257)	*Positive studies:* (258) *Negative studies:* (259)
Abnormal cytokine production	*Positive studies:* increased pro-inflammatory cytokines (e.g., IL-1A, IL-17a, tumor necrosis factor-α) and “anti-inflammatory” cytokines (e.g., IL-1 receptor antagonist, IL-4, and IL-13) (260–273). Increased cytokines seen particularly in the first 3 years of illness (260, 274). Systematic review finds correlations of specific cytokine elevations to specific symptoms (275). T_*H*_2 cytokines may be elevated relative to T_*H*_1 cytokines (266). Spinal fluid cytokine levels also reflect inflammation with a T_*H*_2 pattern (276). *Negative studies*: (277–283)	*Positive studies:* increased levels of certain pro-inflammatory cytokines, including IL-1ß, IL-6, and TNF (207, 284–292). However, reduced levels of several pro-inflammatory cytokines/cytokine receptors and chemokines (IL-18, TNF-RII, MCP-1/CCL-2) (293). SARS-CoV-2 spike protein induces production of proinflammatory cytokines by microglial cells (294). *Negative studies:* (295)
Cytokine levels correlate with severity of symptoms	*Positive studies:* (271)	*Positive studies:* (296) *Negative studies:* (295)
Increased levels of circulating immune complexes	*Positive studies:* (245, 297) *Negative studies:* (298)	
Increased numbers of activated CD8+ cytotoxic cells	*Positive studies:* (252, 299–301)	*Positive studies:* (258, 284, 286, 288)
T cell exhaustion in long-term illness	*Positive studies:* (260, 274, 302)	*Positive studies:* (258)
B cell abnormalities	*Positive studies:* increased numbers of CD21+, CD19+, activated CD5+, and CD24+ B cells (243, 303–305). Gene expression pattern suggesting impaired B cell differentiation and survival (306). Increased levels of B lymphocyte activating factor (307). Increased frequency of HLA alleles associated with autoimmunity (DQB1, KIR3DL1, and KIRDS1) (308–310). Antigen-driven clonal B cell expansion (311, 312).	*Positive studies:* (288) *Negative studies:* (259, 286)
Increased levels of autoantibodies	*Positive studies:* multiple polymorphisms linked to autoimmunity (313). Increased antinuclear antibodies (314, 315), anticardiolipin and antiphospholipid antibodies (208, 316, 317), antineuronal antibodies (318), antiganglioside antibodies (209) and antiserotonin antibodies (209). Autoantibodies against CNS and autonomic nervous system targets correlate with the presence and severity of symptoms (319).	*Positive studies:* in acute COVID, there are many autoantibodies, including to neural/autonomic targets (320). Autoantibodies also found in Long COVID (219, 321–323). Autoantibodies can be functionally active and correlated with symptoms (219, 296, 321, 322, 324, 325). Some autoantibodies may be associated with decreased risk of Long COVID (326, 327). Some autoantibodies that are clearly involved in the pathophysiology of acute COVID do not appear to have a role in Long COVID (328).
Increased apoptosis of white blood cells	*Positive studies:* increased apoptosis (329–331). Upregulation of apoptosis-associated genes or microRNAs (332–336). *Negative studies:* (337, 338)	*Positive studies:* SARS-CoV-2 spike protein induces apoptosis of microglial cells (294).
Characteristic histocompatibility antigens (HLA)	*Positive studies:* HLA-DQ1 and -DQ2 (308, 339, 340). *Negative studies:* (341)	
Alterations in leukocyte transcriptome		*Positive studies:* compared to recovered COVID-19, significant differences in genes linked to cell cycle, CD4+ cells, genes related to monocyte and myeloid cell function (259).
Increased numbers of T regulatory cells	*Positive studies:* (342, 343) *Negative studies:* (252)	*Positive studies:* (258)
Mast cell activation syndrome	*Suggestive studies:* (136, 344, 345)	
Pattern of micro-RNA expression implicating inflammation	*Positive studies:* (346)	
Distinctive methylome implicating inflammation	*Positive studies:* (347)	
**SARS-CoV-2**		
Evidence of reservoirs of SARS-CoV-2 nucleic acid and antigen in multiple tissues	Not applicable	*Positive studies:* (11, 348–353)
**Non-SARS-CoV-2 viral agents**		
Reactivation of latent herpesviruses	*Positive studies:* (354–359) *Negative studies:* (360)	*Positive studies:* in acute COVID-19, reactivation EBV is frequent (361) (or only modest) (362, 363), as is reactivation of HHV-6, HHV-7 and CMV (361, 363, 364). *Positive studies in Long COVID:* (7, 365, 366)
**Gut microbiome studies**		
Proinflammatory gut and oral microbiome with dysbiosis	*Positive studies:* (367–370)	*Positive studies (gut microbiome):* (371–373) *Positive studies (oral microbiome)* (372)
**Evidence of accelerated senescence**		
Various markers of senescence, in various cell types, including senescence-associated secretory phenotype (SASP) and shortened telomere length	*Positive studies:* (374, 375)	*Positive studies:* (375–377)

In addition, as summarized in Table 5, differences in the frequency of several reactivated herpesviruses, and differences in the gut microbiome, distinguish patients with these two illnesses from comparison/control groups.

### Metabolic abnormalities

Beginning in the 1990s, evidence began to accumulate indicating that in people with ME/CFS who experienced a lack of “energy,” a contributing factor might be a cellular failure to generate and utilize adenosine triphosphate (ATP). As summarized in Table 6, considerable evidence has since emerged in support of that hypothesis. The ability to generate energy from multiple sources is impaired: from fatty acids, amino acids, glucose and oxygen. Moreover, similar evidence is emerging in people with Long COVID.

**TABLE 6:** Metabolic abnormalities.

Finding	ME/CFS	Long COVID
**Energy generation**		
Reduced ATP from fatty acids	*Positive studies:* (378–389) In healthy people, acylcarnitine levels and, hence, ß-oxidation of fatty acids (main source of ATP during aerobics) rises with exercise. But acylcarnitine falls with exercise in ME/CFS (390). CD4+, CD8+, and NK cells rely more on fatty acid oxidation for generating ATP in people with ME/CFS vs. controls (302).	*Positive studies:* impairment of ß-oxidation of fatty acids at rest (293) and during exercise (391). Abnormal levels of free- and carnitine-conjugated mono-, poly-, and highly unsaturated fatty acids (392). Patients with more depleted levels of γ-linolenic acid (GLA) and eicosapentaenoic acid (EPA) during recovery from acute COVID-19 are more likely to develop Long COVID (393). Depletion of EPA impairs vasodilation, worsens oxidative stress and enhances inflammation, making it a plausible contributor to the pathogenesis of Long COVID (394).
Reduced ATP from amino acids	*Positive studies:* reduced levels of multiple amino acids (390). Reduced ability to generate ATP from amino acids (384–386, 388, 395–403).	*Positive studies:* elevated taurine, glutamine/glutamate ratio, and kynurenine/tryptophan ratio in Long COVID vs. people fully recovered from COVID-19 (404). SARS-CoV-2 infection of intestinal organoids leads to decreased absorption of tryptophan, tyrosine, phenylalanine, precursors to neurotransmitters (405).
Reduced ATP from glucose via tricarboxylic acid (TCA) cycle	*Positive studies:* (400, 406, 407) This defect may exist prior to the “infectious-like” event which is followed by ME/CFS (408).	*Positive studies:* (392)
Reduced ATP from glucose via glycolysis	*Positive studies:* (253, 274, 398, 409) *Negative studies:* (410)	
Mitochondrial/oxidative phosphorylation abnormalities (reduced ATP production from oxygen)	*Positive studies:* (335, 411–416)	
Abnormalities in nucleotides central to energy metabolism	*Positive studies:* (385, 388, 407, 409) This defect may exist prior to the “infectious-like” event which is followed by ME/CFS (408).	
**Hypometabolic state**		
Hypometabolic state: reduced metabolite levels in blood	*Positive studies:* (103, 104, 384, 390)	*Positive studies:* no studies of metabolite levels in blood reported. Multiple reports of brain hypometabolism on imaging (see Table 4).
**Redox imbalance**		
Increased levels of pro-oxidants	*Positive studies:* increased levels of peroxides and superoxides which correlate with the severity of symptoms (417). Increased levels of isoprostanes, both at rest and after exercise (331, 418, 419).	*Positive studies:* SARS-CoV-2 spike protein induces oxidative stress in microglial cells (294). Multiple markers of oxidative stress seen in people with Long COVID (420, 421). RCT of vitamin C (an antioxidant) improved symptoms (422, 423).
Decreased levels of antioxidants	*Positive studies:* decreased levels of glutathione (408) and α-tocopherol (424). Elevated levels of thiobarbituric acid reactive substances, or TBARS (170, 425, 426), that correlate with severity of symptoms (170, 426). This defect may exist prior to the “infectious-like” event which is followed by ME/CFS (408).	*Positive studies:* (421) Symptom severity correlates with markers of oxidative stress (421). Glutathione levels low in brain (150).
Increased nitrosative stress	*Positive studies:* increased levels of inducible nitric oxide synthase (iNOS), nitric oxide (NO), peroxynitrite, and nitrate (427–429). Increased nitrosative stress is further augmented by exercise (430), and may be secondary to increased production of NFκB (431) and impaired autophagy (432).	*Positive studies:* an RCT of L-arginine improved symptoms attributed to endothelial dysfunction (423).
Brain oxidative stress	*Positive studies:* magnetic resonance spectroscopy reveals elevated levels of ventricular lactic acid and other markers of oxidative stress (204, 433, 434).	
**Kynurenine pathway abnormalities**		
L-tryptophan levels	*Elevated:* (435, 436)	*Reduced:* (392, 437, 438)
Kynurenine/tryptophan ratio	*Reduced* in an ME/CFS-like state induced by interferon therapy (439).	*Increased:* (437, 438)

In addition to energy metabolic deficits, evidence of a systemic hypometabolic state (also manifest in the brain, Table 4), abnormalities in redox balance, and abnormalities in the kynurenine pathway have emerged in both illnesses, as summarized in Table 6. Later, we discuss briefly how abnormalities in redox balance may have bidirectional connections to abnormalities in the immune response and energy generation.

### Cardiopulmonary and vascular abnormalities

Although less extensively studied than neurological, immunological, infectious or metabolic abnormalities, a growing number of cardiopulmonary abnormalities have been identified, as summarized in Table 7. The most well documented abnormalities are diminished exercise capacity on exercise testing, particularly when a second exercise test is performed 24 h after the first; reduced ventilatory efficiency; and endothelial dysfunction (particularly in Long COVID but also in ME/CFS). Some of the metabolic abnormalities noted in Table 6 are provoked by exercise, and thus there is some overlap between Tables 6, 7.

**TABLE 7:** Cardiopulmonary and vascular abnormalities.

Finding	ME/CFS	Long COVID
**Cardiopulmonary exercise testing**		
Reduced peak VO_2_ (diminished exercise capacity)	*Positive studies:* reduced on initial CPET (180), and worsened when CPET is repeated 24 h later (440, 441), as confirmed by several meta-analyses (442–444). *Negative studies:* one author finds evidence this is due to failure to control for deconditioning (445, 446)	*Positive studies:* (391, 447–464) Diminished exercise capacity present more often in those most severely ill during acute infection but occurs even in those only mildly or moderately ill with acute infection (449, 452, 462, 465). *Negative studies:* (466–470) Systematic reviews and meta-analyses have judged the literature to be sufficiently variable in methodology that it is hard to extract a clear message (459, 471).
Increased VE/VCO_2_ slope (reduced ventilatory efficiency)	*Positive studies:* (180, 446)	*Positive studies:* (447, 449–452, 455, 457–459, 463, 464, 468) *Negative studies:* (453, 454, 461, 466, 469)
Exercise reveals impaired systemic oxygen extraction, impaired venous return, possible arterial-to-venous shunting, and ventilatory inefficiency	*Positive studies:* (180, 446)	*Positive studies:* (464)
Increased lactic acid in muscle with exercise	*Positive studies:* (472, 473)	*Positive studies:* (391)
Increased oxidative and nitrosative stress with exercise	*Positive studies:* (197, 425, 430), accompanied by multiple objective metabolic abnormalities (474).	
Worsened symptoms after exercise	*Positive studies:* (39)	
Distinctive epigenetic changes induced by exercise	*Positive studies:* (475)	
Distinctive inflammatory exosome production during exercise	*Positive studies:* (476)	
Gut bacteria and bacterial antigens enter circulation during exercise	*Positive studies:* (477)	
Dysautonomia reflected in heart rate response to posture and movement/exercise	*Positive studies:* meta-analysis (122); chronotropic incompetence found (127) (see Table 4, autonomic dysfunction)	
**Cardiac chamber and vascular space blood volume**		
Reduced preload (venous return to the right heart)	*Positive studies:* (180, 478)	*Positive studies:* (464)
Reduced blood volume	*Positive studies:* (479–484)	
Cardiac chamber volume reductions at rest	*Positive studies:* MRI-documented reduction in stroke volume and end-systolic and end-diastolic volumes. (485)	
**Endothelial dysfunction**		
Endothelial dysfunction (ED)	*Positive studies:* (486–495) Endothelial dysfunction involves both macrovascular and microvascular vessels (495).	*Positive studies:* microvascular injury and ED are cardinal features of acute COVID-19 in humans (10, 496) and in non-human primates (497) and may contribute to the greatly increased risk of cardiovascular diseases for the next several years (12) Impaired endothelium-dependent flow-mediated dilation and prothrombotic proteins often persist after severe acute COVID-19 (498–500). ED greatest in those who were most ill with acute COVID-19 (501). Markers of accelerated angiogenesis found in Long COVID and accurately distinguish Long COVID from acute COVID and from healthy controls (502). Neutrophil extracellular traps (NETs) present more often in Long COVID (323). Serum contains factors that promote angiogenesis and inhibit endothelial relaxation (503–505). ED demonstrated by impaired reactive hyperemia index and elevated endothelin-1 (506). Transcranial Doppler demonstrates impaired cerebral perfusion 300 days after initial infection, even in people with mild acute illness, compared to controls (507). RCT finds antioxidant treatment (L-arginine + vitamin C) directed at improving endothelial function significantly improves flow-mediated dilation and fatigue (508).
**Platelet abnormalities and coagulopathies**		
Platelet abnormalities/coagulopathies	*Positive studies:* hyperactivated platelets and microclots (494).	*Positive studies:* presence of fibrin amyloid microclots that impair microcirculation (504, 509) that may be more severe in people with more severe disease (510). Differences in genes linked to platelet function (259).

## Discussion

### Similarities and differences in symptoms

As summarized in Table 3, most of the symptoms reported with ME/CFS and Long COVID are similar. Decreased smell and taste, rash and hair loss are more likely in Long COVID than ME/CFS; this may reflect pathology induced specifically by SARS-CoV-2 (543).

Not only is a common core of symptoms shared by ME/CFS and Long COVID: these same symptoms also are also reported following multiple infectious illnesses (544) and major *non-infectious* injury such as post-critical illness syndrome or post-intensive care unit syndrome (545, 546), including heat stroke (547).

### Similarities and differences in underlying biology

In this review we have compared what is known about the underlying biology of ME/CFS and Long COVID. Both clearly are systemic illnesses involving multiple organs and physiological systems. Long COVID is triggered by infection with SARS-CoV-2. ME/CFS often is triggered by an “infectious-like” illness. We think it is unlikely that ME/CFS is triggered by a single, novel infectious agent: more likely, it represents a dysfunctional response to infection with any of multiple agents, as recently described (544).

As summarized in this review, both illnesses share abnormalities involving the central and autonomic nervous systems, the immune system, reactivation of latent infectious agents (primarily herpesviruses), the gut microbiome, energy metabolism, a hypometabolic state, redox imbalance, and various cardiac, pulmonary and vascular abnormalities.

Many of these abnormalities bidirectionally influence each other. This creates the potential for multiple, self-reinforcing “vicious” pathophysiological cycles that could lead to persisting illness (548, 549). It also means that the precipitating event, which sets in motion those vicious cycles, may be different in one person with the illness from the precipitating event in another person.

In summarizing what is known about biological abnormalities in both illnesses, we have cited both the “positive” studies that find an abnormality and the “negative” ones that do not. These citations allow readers to assess the strength (or weakness) of a reported finding.

As shown by the tables, some reported findings are supported by robust evidence—confirmatory reports from multiple laboratories: even though there may be some dissenting reports, the preponderance of the evidence supports the finding. The tables also identify abnormalities that are not supported at this time by robust evidence and require future investigation.

### Post-infection/post-injury syndrome

The similar symptoms and pathology of ME/CFS and Long COVID raise the question of whether these disorders represent just two examples of a broader illness in which symptoms occur because they are generated by a carefully orchestrated, stereotyped, multi-system response to infection and injury.

Why might such a carefully orchestrated group of symptoms be generated following infection or injury? We speculate, as have others (550–552), that these symptoms are generated because they lead to metabolic reprogramming (553) as well as to behavioral changes that reduce non-essential, energy-consuming activities—thereby maximizing the amount of energy available to facilitate recovery. Fatigue, myalgia and orthostatic intolerance, for example, lead to reduced physical activity, redirecting energy stores to eradicate infection and heal tissue injury.

Such a carefully orchestrated response to vital threats exists throughout the animal kingdom. The best studied examples are hibernation and torpor in “higher” animals (554–556), and the larval state of dauer in the worm *C. elegans* (557–559).

Dauer, hibernation and torpor all involve “abnormal” innate immune responses, redox imbalance, increased glycolysis, decreased aerobic respiration, and possibly even alteration in the organisms’ microbiome. They also involve orchestration by the autonomic nervous system (and its counterparts in the nervous systems of more primitive organisms). Thus, these well-recognized “hunkering down” mechanisms all exhibit similarities to the emerging pathophysiology of ME/CFS and Long COVID, as summarized in this review.

## Conclusion

The goal of this report is to provide a road map to the state of knowledge about the underlying biology of ME/CFS and Long COVID. The often-similar findings suggest that insights into each disorder will have implications for the other. They may also enhance our understanding of evolutionarily preserved biological responses that fight infection and heal injury. We urge that investigators studying the underlying biology of Long COVID take note of the robust findings in ME/CFS that have not yet been investigated in Long COVID: given the many similarities in the underlying biology of the two illnesses, it is likely that pursuing such abnormalities in Long COVID will prove instructive.

Research into the pathophysiology of these responses has the potential to lead to new strategies for reducing the morbidity of ME/CFS and Long COVID, and of similar illnesses that can follow a variety of infections and non-infectious traumatic injury.

## Author contributions

AK and WL conceptualized and wrote the manuscript. Both authors contributed to the article and approved the submitted version.
